# Padova Emotional Dataset of Facial Expressions (PEDFE): A unique dataset of genuine and posed emotional facial expressions

**DOI:** 10.3758/s13428-022-01914-4

**Published:** 2022-08-24

**Authors:** A. Miolla, M. Cardaioli, C. Scarpazza

**Affiliations:** 1 Department of General Psychology, University of Padua, Padua, Italy; 2 Department of Mathematics, University of Padua, Padua, Italy; 3 GFT Italy, Milan, Italy

**Keywords:** Facial expressions, Genuine emotions, Posed emotions, Emotion dataset

## Abstract

Facial expressions are among the most powerful signals for human beings to convey their emotional states. Indeed, emotional facial datasets represent the most effective and controlled method of examining humans’ interpretation of and reaction to various emotions. However, scientific research on emotion mainly relied on static pictures of facial expressions posed (i.e., simulated) by actors, creating a significant bias in emotion literature. This dataset tries to fill this gap, providing a considerable amount (*N* = 1458) of dynamic genuine (*N* = 707) and posed (*N* = 751) clips of the six universal emotions from 56 participants. The dataset is available in two versions: original clips, including participants’ body and background, and modified clips, where only the face of participants is visible. Notably, the original dataset has been validated by 122 human raters, while the modified dataset has been validated by 280 human raters. Hit rates for emotion and genuineness, as well as the mean, standard deviation of genuineness, and intensity perception, are provided for each clip to allow future users to select the most appropriate clips needed to answer their scientific questions.

## Introduction

Facial expressions represent an innate and automatic behavioral component of emotional and social communication (Motley & Camden, 1988; Jack et al., 2016; Zloteanu et al., 2018; Darwin, 1872). Emotional facial expressions, in particular, have a communicatory function that conveys specific information to the observer (Andrew, 1963; Darwin & Prodger, 1998; Ekman et al., 1969; Jack et al., 2012; Jack & Schyns, 2015). For example, an expression of happiness through a smile, in response to a particular behavior, increases the probability that the action will be repeated in the future, differently from an angry or sad face (Motley & Camden, 1988). In this sense, the nature and the interpersonal function of the emotional facial expressions conveys a message that predicts different social outcomes (Darwin, 1872; Ekman, 1972). It is precisely for this reason that accurately deciphering what someone is trying to communicate through facial expression is extremely important in day-to-day social interactions (Johnston et al., 2010). Importantly, emotions conveyed by faces can change under several parameters. We can display different varieties of expressions: some intense and sustained, while others are subtle and fleeting (Ambadar et al., 2005). One of the most high-level and critical communication features is related to the perception of authenticity of the emotion expressed (Lu et al., 2020; Rooney et al., 2012). In fact, we can express emotions spontaneously, triggered by real circumstances (i.e., “event-elicited”) (Dawel et al., 2017). For example, someone might be scared because he is genuinely afraid of a snake or be sad because of the loss of a loved one. Conversely, we can deliberately feign or pose emotions in the absence of a congruent underlying context in order to receive adaptive advantages. These expressions reflect the strategic intent of the sender in the absence of felt emotions (Ekman & Rosenberg, 2005).

For example, pretending to be sad can be a useful strategy to take advantage of a perceiver’s reciprocal kindness or compensatory behavior in response (Reed & DeScioli, 2017). The endogenous nature of emotional experiences (i.e., genuine or posed) completely changes the observer’s perception and reaction. In social interactions, perceiving others’ emotional reactions as genuine might promote social interaction and increase the expresser’s trustworthiness (Reed & DeScioli, 2017). For example, Johnston et al., (2010) showed how genuine (or spontaneous) smiles make perceivers more cooperative than posed smiles. In psychotherapy, therapists’ genuineness, authenticity, and honesty their credibility, which is essential for promoting therapeutic alliance and patients’ trust (Lu et al., 2020; Jung et al., 2015; Dowell and Berman, 2013; Schnellbacher & Leijssen, 2009). Furthermore, in movies, the perception of realism in the actor’s performance may promote a more emphatic mechanism and a more emotional contagion of the perceivers (Rooney et al., 2012). From a neuropsychological point of view, it has also been argued that genuine and fake emotions may recruit different components of emotional contagion (Manera et al., 2013). For example, there is evidence that genuine smiles are associated with the experience and physiological activations of positive emotions, while posed ones with the experience and physiological activation of negative emotions (Davidson et al., 1990; Ekman et al., 1990; Soussignan, 2002).

Despite this evidence, only recent studies on perception of emotions conveyed by faces used stimuli depicting genuine facial expressions so far (Künecke et al., 2017; Vergallito et al., 2020; Zloteanu et al., 2018; McLellan et al., 2012). Indeed, the vast majority of previous research investigating perception of facial expressions have focused on posed (or fake) emotions (Dawel et al., 2017; Tcherkassof et al., 2013), raising serious doubts about the ecological validity of these studies (Tcherkassof et al., 2013; Barrett et al., 2019; Russell, 1994; Wallbott & Scherer, 1986; Zuckerman et al., 1976; Wallbott, 1990). Spontaneous/genuine and posed/fake emotional expressions differ in their temporal and morphological characteristics, such as duration, intensity, and asymmetry (Cohn & Schmidt, 2003; Ekman, 1997; Sato & Yoshikawa, 2004; Valstar & Pantic, 2010; Wehrle et al., 2000; Yoshikawa & Sato, 2006). Indeed, posed emotions display stereotypical and exaggerate facial configuration that is rarely met in real life (Barrett et al., 2019). On the other side, spontaneous emotions in real life are usually less intense, more subtle, and more difficult to detect (Tcherkassof et al., 2013; Dawel et al., 2017). As a result of the strict focus on prototypical posed facial expressions, it is evident that researchers may have underestimated the considerable differences between spontaneous and posed emotional facial expressions. It is thus still not known whether our knowledge of processing of emotions conveyed by faces is biased by the fact that studies have been conducted using stimuli displaying stereotypical emotions. This important bias makes unknown whether the results on emotions perception from faces so far available within the scientific literature are driven by the (un)conscious perception of the non-authenticity of the perceived emotions. Even more importantly, it is not known whether results obtained using posed emotions are generalizable to genuine emotions.

These research questions are still unanswered also because the scientific community is still devoid of a validated dataset of stimuli including both genuine emotions from the same actors. Although some datasets including genuine and posed emotions seem to be present in literature (please see Krumhuber et al., 2017 for a review), their usefulness is limited as the emotions expressed are elicited by methods that limited the spontaneity of the subjects’ facial displays (e.g., subjects were aware of the aim of the studies, thus creating a barrier in the elicitation of spontaneous emotions) and actors were not asked to rate the genuineness of the expressed emotions (Kulkarni et al., 2018; Cheng et al., 2018; Novello et al., 2018) with the consequence that emotions displayed in these dataset are not perceived as genuine by the observers (Dawel et al., 2017) In addition, these dataset are not validated (Kulkarni et al., 2018; Cheng et al., 2018), or do not include posed emotions, preventing the comparison between genuine and posed emotions (O’Toole et al., 2005; Sebe et al., 2007), or emotions are displayed only through static pictures (Dawel et al., 2017; Novello et al., 2018). Finally, these dataset includes only few emotions (i.e., McLellan et al., 2010includes only happy and sad expressions).

The current work aims to enrich future research of emotions providing the scientific community with a new dataset of emotional stimuli conveyed by faces, that includes a considerable amount of both spontaneous and posed emotional facial expressions of the six basic emotions. We called this dataset Padova Emotional Dataset of Facial Expressions (PEDFE). The contributions of the current research are mainfold: first, PEDFE includes a considerable amount of emotional clips for both spontaneous and posed emotions. The same emotion is displayed genuinely and posed for each participant, allowing a direct comparison (i.e., intra-subject and between-subject) between these two ways to convey emotions through facial expressions. Second, the elicitation protocol uses a multimodal sensorial perception to elicit emotions as natural as possible, avoiding any restrictions or influences by the researcher (please see Section “Emotion elicitation procedure”). To the best of our knowledge, the current emotion elicitation protocol has more tasks (i.e., 15) than the other reported methods. Third, all stimuli were validated by asking subjects to rate each clip according to the emotion, genuineness, and intensity of the facial expression perceived. This is an essential step in creating emotional datasets that most of the datasets displaying genuine and posed emotions neglected. Last, the enhanced version of the PEDFE (see Sections “Dataset creation” and “Results”) qualifies as the first spontaneous dataset displaying only the face, removing all distracting variables from the background (e.g., hair, clothes, color of the background, ecc), and providing several advantages in research (Davies et al., 1994; Minami et al., 2018; Tsao & Livingstone, 2008; Xu et al., 2017).

## Dataset creation

### Participants selection procedure and compliance with ethical standards

Fifty-seven participants, aged between 20 and 30 years, took part in the experiment. Participants were randomly assigned to one of the two settings with the proportion 1:2 (please see Section “Experiment setup”). The sample was enrolled using an advertisement on the University Website and were compensated for their participation. Participants signed an informed consent before the beginning of the experiments. After reading this informed consent, they were still unaware of the purpose of the study and were unaware of being filmed. The participants were informed that they had the right to quit the experiment and withdrew their consent at any time. At the end of the session, participants were debriefed, and the study’s real aims were revealed. They were also told they were recorded. One participant withdrew her consent, and her clips were permanently removed from the database. The experimental procedure and the emotional elicitation protocol submitted to the participants and described in the following paragraphs were approved by the Ethics Committee of the University of Padua (Protocol number: 2917). The participants’ video recordings were included in the database only after they signed a written consent to use their videos for research purposes.

### Experiment setup

The aim of the experimental procedure was to record spontaneous (i.e., stimulus elicited) emotions of participants while they watched emotional video or were performing simple tasks. For this reason, participants were left alone in an experimental room to decrease the possibility that embarrassment and social inhibition could affect the spontaneity of expressed emotion, impacting on the overt manifestation of emotions. The doors and windows were kept shut during the entire protocol to avoid external interference and allow participants a more in-depth emotional excursion during the tasks. Participants were set about one meter in front of a Lenovo ThinkPad T490. As it is known that awareness of the experimental aim can interfere with the spontaneity of overt emotional expression (Happy et al., 2015; Sebe et al., 2007), participants were unaware of the purpose of the experiment. For this reason, a cover story was created. In particular, participants were told they have to rate emotional valence of the videos, as already did for a previous study (Happy et al., 2015). They were also told that, in order to accurately assess emotions, they had to try to get immersed in the viewing experience and feel free to experience their emotions. Moreover, subjects were allowed to sit at their ease without any other restrictions inside the experimental room to avoid possible suspects or limit the emotions’ naturalness.

**Fig. 1:**
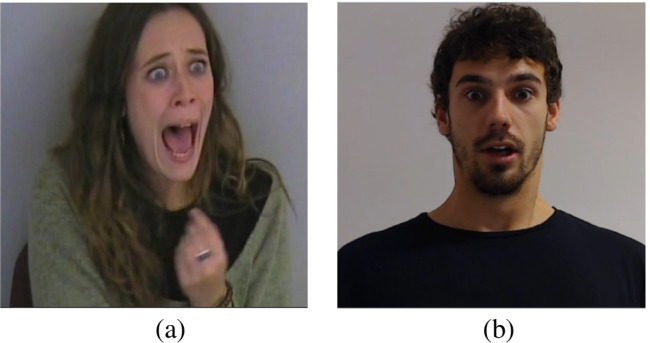
Examples of fear expressions for the two settings

The same protocol was submitted in one of the two following modalities in order to enrich the database with different viewing angles. Overall, 21 and 35 participants were assigned to the first and second setting respectively. The first setting was created based on the well-known assumption that awareness of being filmed might impacts on spontaneity of overtly expressed emotions. Thus, in this first setting, a hidden camera placed at the right room’s top angle was used. Participants were thus totally unaware of being recorded, preserving the emotional reactions’ spontaneity. The clips were recorded with a AW-HE40HWEJ–Panasonic at a distance of at least 2 meters, with an angular size of 20^∘^, varying in accordance with the head movements of subjects. The second setting was thought with the aim to create video depicting the participants on a frontal view. For this reason, in the second setting, a Logitech C920 HD Pro Webcam, Full HD 1080p/30fps, was placed at the top of the computer screen used for the tasks. In this setting, to preserve the subjects’ expressions’ spontaneity, participants were told that the recording was necessary to study the eye movements and pupil dilatation while performing the valence rating task. The two experimental setups guarantee more options to the experimenter who will use the emotional stimuli by having the same emotions (both spontaneous and posed) with a front and a lateral view (see Fig. 1).

### Pilot subjects

Before starting the data collection, we run 10 pilot subjects to identify the most appropriate and effective strategies to elicit genuine emotions, as we needed emotions not only to be felt, but also to be overtly expressed by the face. Genuine emotions are very difficult to be elicited as they are sometimes considered private, and some individuals experience more difficulties to overtly manifest them. Furthermore, in an experimental setting, people might feel embarrassed or repressed in fully expressing their emotions. This is mostly true for some emotions, like anger, which is often not socially acceptable, and sadness, which is often felt as personal and embarrassing. The pilot protocol was also run with the very same 10 participants to identify the potential difficulties in genuine emotion elicitation. The trial subjects were not recorded, and data were not collected as the only purpose of the pilot subjects was to identify the most effective emotion elicitation strategy. We adapted the protocol (i.e., the order of the tasks) according with the emotions overtly manifested by the subjects and the feedbacks we received from them during the post section debriefing. We realized that some emotions were quite simple to elicit, for instance happiness. Other emotions, like fear, sadness, and anger, were very difficult to elicit. Participants told us that: i) they have difficulties in expressing fear; ii) they felt sadness but they felt embarrassed to express it and they did not have the time to feel completely immersed in the emotional experience; iii) they felt anger but were able to control it (even if we explicitly asked them not to try to control their emotions). In addition, participants also suggested that they felt disgusted by different things. Since our aim was exclusively to find effective strategies to elicit all the emotions in each individual, we thus had to adapt the protocol adding tasks in order to be sure to capture at least one genuine emotional expression for each participant. For this reason, it was not possible to have the same number of emotional inducing task for each emotion. Thus, more than one stimuli were chosen for each emotion (but happiness) to enhance the probability of eliciting the target emotion and collecting more samples of displayed emotions for each subjects. For example, for sadness, we used five tasks to trigger and collect sad facial expressions. This choice was due to the peculiar characteristics of sadness, which is associated with loss of muscular tone and a focus on inner thoughts and feelings (Ekman & Friesen, 2003; Izard, 1991) that make sadness more difficult to detect. For disgust, as pilot subjects declared to be disgusted by bad smells, we added a task where they had to smell stinky solution. For anger, as participants told us to have difficulties in overtly express it, we provided them with a desktop punching ball, to favor the overt expression of anger.

At the end of the pilot, ten additional subjects were asked to identify the emotions they felt during the tasks. As the emotions felt by each pilot subject correspond to the ones the emotion elicitation task was initially selected to elicit, the protocol was confirmed.

### Emotion elicitation procedure

Spontaneous emotional reactions were elicited with a multimodal protocol described in Table 1. Emotions were mostly triggered by watching emotion-inducing videos, which resulted to be the most effective stimuli for evoking emotional responses (Carvalho et al., 2012). The clips were selected from different stimuli that have been used for similar studies (Rottenberg et al., 2007), and from other sources such as international films, commercial spots, and YouTube clips. The length of the clips did not exceed 5 min according to the recommended size of the emotional video (Rottenberg et al., 2007). The emotions were not only elicited through passive elicitation by watching emotion-inducing videos. For example, anger was also triggered by using a rage game, well-tested stimuli to provoke anger, in which the emotion was elicited as a result of the encoder actively engaging with the game (Sneddon et al., 2011). Indeed, the typology of these games was designed to make the task very difficult to purposely increase a high level of frustration and anger to the players. As, in pilots trails, we found that anger is often repressed, we provide participants with a desktop punching ball. Finally, as olfactory stimuli can reliably elicit disgust and have been resulted in very efficiently in previous studies (Zhang et al.,^2016^; Hayes et al., 2009a, 2009b), an unpleasant odor was presented to the subject to induce a disgusting feeling.

After the end of each task, participants were asked to identify the emotion they experienced/felt within the six basic emotion and neutral. They were also given the possibility to report if they felt an emotion that was not included within the six basic ones. Furthermore, besides identifying the emotion felt, they were also asked to rate how much the emotion they felt was genuine on a Likert scale ranging from –7 to + 7 where –7 corresponded to “completely not genuine” and + 7 corresponded to “completely genuine”, according with previous literature (Dawel et al., 2017). Finally, participants rated the intensity of the emotions experienced during the tasks on a Likert scale ranging from 0 (“Emotion not felt/No intensity”) to 9 (Emotion felt very intense/Very Strong Intensity”) (Dawel et al., 2017).

When the multimodal emotion elicitation protocol was successfully concluded, participants were asked to pose the six basic emotions multiple times, modulating the intensity of the posed emotions. In particular, participants were asked to pretend to feel a target emotion and to pose that emotion for at least 15 second different times trying to modulate its intensity. During this task, they were also asked to use the same objects they used during genuine emotion elicitations (i.e., punching ball and olfactory stimuli). After the end of each trial, they were debriefed about the emotions they felt and expressed and all of them confirmed they did not felt any kind of emotions, and thus that emotions expressed are to be considered not genuine as they were only posed but not felt.

**Table 1:** Multimodal protocol for spontaneous and posed emotion elicitation. Tasks are presented in this table in the same order they were presented to participants

Task	Emotion	Activity	Description	Length
T1	Sadness	Watch a VIDEO: Death of Mufasa, from the Lion King^1^	The clip displayed the saddest part of the movie, when Mufasa dies because of Scar, and the touching reaction of Simba.	02:42 min
T2	Sadness	Disney Pixar Up^2^	The scene where Ellie and Carl are shown. Their relationship is being shown as time passes from their wedding to Ellie’s death.	04:21 min
T3	Sadness	“Giving without expecting anything in return is the best communication”^3^	Spot for Telecom in Thailand. The story is about kindness rewarded over the course of 30 years.	03:08 min
T4	Sadness	“Love is a gift”^4^	It’s a short film about a man counting down the days to Christmas so he can continue his yearly tradition sparked by a tragic moment from the past.	02:25 min
T5	Sadness	“Edeka 2015 Christmas Commercial”^5^	Edeka’s holiday commercial reminds people of the important things in life in a tragic piece of storytelling.	01:30 min
T6	Surprise	The Invisible Gorilla^6^	An experiment in Change Blindness.	01:00 min
T7	Happiness	When Harry met Sally	This is a classic and funny part to a very good movie. The restaurant/deli scene where Sally fakes an orgasm to prove a point.	02:46 min
T8	Surprise	Colour Changing Card Trick^7^	An experiment in Change Blindness.	02:43 min
T9	Anger	Flappy Bird^8^	A so-called “Rage game”, namely a game while gaming and can’t accomplish your goal whatever that is, and you get random from your lack of success.	05:00 min
T10	Fear	Scare Jump^9^	A so-called jump scare, namely a game intended to scare the audience by surprising them with an abrupt change in image, co-occurring with a frightening sound.	04:00 min
T11	Anger	Abused dog in a metro	The clip showed the abuse of a dog, beaten by his owner on a public metro.	03:00 min
T12	Fear	Scare jump horror clip	A classic horror clip aimed to scare participants with frightening scenes and spectral sounds.	02:28 min
T13	Disgust	Pimples squeezing^10^	Disgusting huge and ingrown pimples are squeezed in the clip.	05:00 min
T14	Disgust	Stinky potion	A solution characterized by an unpleasant smell that causes a strong reaction of disgust.	01:00 min
T15	-	Simulation Session	Participants were asked to pose each emotion as authentic as possible for 30 s each, trying to change their intensity. In addition, some pictures taken by PoFA (Ekman, 1976) were provided to participants to help them move particular facial configurations in order to express their emotions adequately.	06:00 min

^1^ https://www.youtube.com/watch?v=URGUQlcAoNUab_channel=larablacklady

^2^ https://www.youtube.com/watch?v=F2bk_9T482g&ab_channel=xXJEashXx

^3^ https://www.youtube.com/watch?v=cLCE9_JHjPE&ab_channel=Mercating

^4^ https://www.youtube.com/watch?v=JHX0btJYcyI&lab_channel=PhilBeastallFilms

^5^ https://www.youtube.com/watch?v=4_B6wQMd2eI&_channel=WIACZO

^6^ https://www.youtube.com/watch?v=0grANlx7y2E&ab_channel=PhillipNorthield

^7^ https://www.youtube.com/watch?v=v3iPrBrGSJM&ab_channel=Quirkology

^8^ https://flappybird.io/

^9^ https://www.gioco.it/gioco/scary-maze

^10^ https://www.youtube.com/watch?v=beAxdoCFnhw&aab_channel=COMPILATIONPOPPINGVIDEOS

### Video extraction

One of the authors (AM), a certified Facial Action Coding System (FACS) coder, extracted the facial expression of emotions present in the recorded videos. The clips’ selection was made considering both the FACS’s criteria (Ekman et al., 1978) and participants’ self-reports.

FACS is a widely used protocol for recognizing and labeling all visually discernible facial movements, called Action Units (AUs). The FACS manual proposes a list of possible combinations of AUs which are typically associated with expression of emotions (Ekman et al., 2002). The current method was used to reliably and accurately extract the emotional facial expressions shown by participants.

In other words, the clips were selected only if the emotion elicited and conveyed by the face (e.g., happiness) matched: i) the target emotion for each task (in order to avoid to include emotions affected by other emotions); ii) FACS criteria (e.g., AU6 + 12) and iii) participants’ self-report (e.g., they declare to have experienced happiness). Conversely, if participants reported having felt constrained and not natural in the emotional experience (e.g., a score of -4 on the genuineness scale), all the expressions associated with the task were removed. Likewise, if participants showed a facial expression associated with an emotion (e.g., a scowl that may reflect anger), the facial change was not selected if participants did not report to have experienced anger. In fact, a scowl is not always a cue of anger but could instead reflect confusion or concentration. This strict procedure aims to reduce the selection of facial expressions that do not convey authentic and spontaneous emotions. Each clip was cut from the onset point (i.e., the first frame when the expression is visible) to the apex (i.e., the period during which the movement was held at the highest intensity reached) of the emotion. Additionally, if the same emotion(s) was repeatedly elicited in a task, the target expressions were selected multiple times, in order to increase the number of clips included in the final dataset and provide more trials of the same emotion for each participant. Lightworks (https://www.lwks.com/), a non-linear editing system (NLE) for editing and mastering digital video, was used to extract the emotional clips’ perfect range frame.

Importantly, the extracted videos include the original audio. We decided to keep the audio to give future researchers using this dataset to have the audio, if needed for their experimental procedure.

## Results

**Fig. 2:**
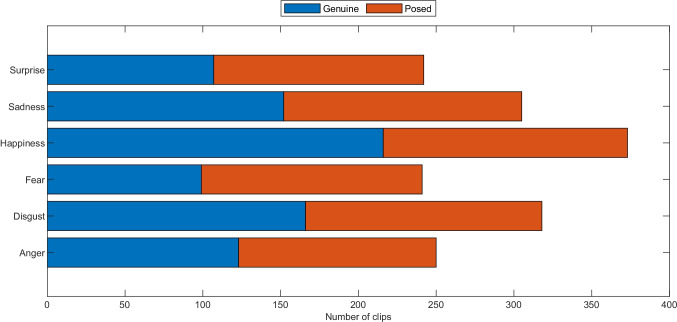
Number of clips before the validation, divided into emotion and type

PEDFE contains clips and static pictures of 56 participants, displaying subtle to full-blown elicitation of different emotions. Overall, the number of emotional clips is 1731 (the exact number clips for each emotion and category are provided in Fig. 2. It is here important to underly that the number of clips do not correspond to the number of subjects multiplied for the number of emotional inducing task for that emotion. As explained within the “pilot subjects” section, many emotions were difficult to be induced, thus, it is possible, to make and example, that some participants overtly manifested sadness only during the observation of one of the five sadness inducing video. It was also likely that participants manifested more than one happy expression during the observation of the same video. Figure 2 thus represents the row number of the emotional expressions we have been able to collect.

**Fig. 3:**
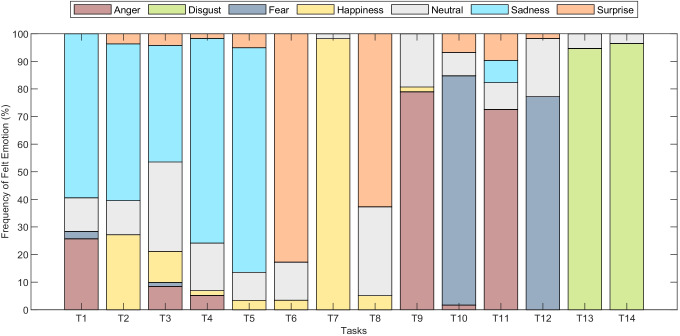
Emotion distribution from self-report for each task

**Fig. 4:**
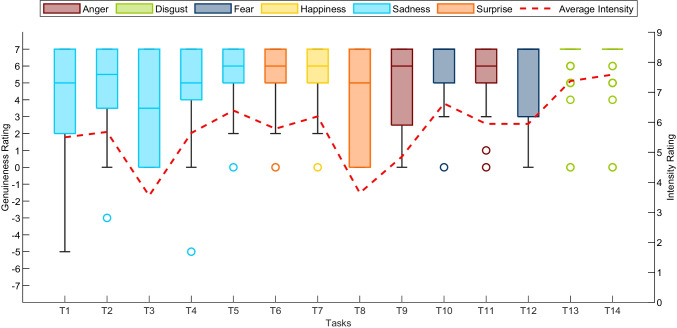
Genuineness and intensity rate distribution for each task. The boxplots of participants’ ratings are provided for the 14 elicitation conditions. The boxplot represents the median and first and third quartile (Q1 and Q3) of the responses. *Error bars* denote the maximum and minimum, defined as *Q*1 − 1.5*IQR* (interquartile range) and *Q*3 + 1.5*IQR*. The *open circles* represent the participants recognized as outliers. The *red dotted line* indicates the average intensity reported for each task

The duration of the facial expressions varied in accordance with the emotion displayed. For example, sad clips last longer (*M* = 5.35*s*; *SD* = 2.92*s*) than other emotions such as happiness (*M* = 2.89*s*; *SD* = 1.25*s*), disgust (*M* = 2.81*s*; *SD* = 1.33*s*) or anger (*M* = 2.92; *SD* = 1.38) because of the gradual evolution of sadness over a longer time-frame. Conversely, emotions like surprise (*M* = 1.94*s*; *SD* = 1.04*s*) or fear (*M* = 1.86*s*; *SD* = 0.92*s*) emerged and disappeared faster, lasting a few seconds at the most (Ekman & Friesen, 2003) The considerable amount of clips (i.e., 1731), as well as the self-reports given by participants, revealed the effectiveness of the elicitation protocol (please see Figs. 3, 4). In fact, most participants reported, on average, to have experienced the emotion that the elicitation tasks aim to do (except for Task 3). This was also confirmed by the intensity reported for each task, reflecting from medium to very high intensity (for the disgust tasks). Furthermore, the genuineness distribution rating revealed the spontaneity and genuineness of the emotional expressions displayed by participants. However, as expected and already reported in similar studies (Happy et al., 2015), the elicitation and recording of facial expressions occurring spontaneous emotional experiences is empirically not easy (Tcherkassof et al., 2013). Indeed, the emotional induction varied according to the subjective perception and sensitivity of the participants. For example, Task 1 (“The Lion King”) was reported as very sad by most of the subjects, while a few experienced fear or anger. Yet, in Task 11 (“Abused dog in a metro”), most participants revealed to have experienced anger. However, others reported sadness, surprise, or even no emotions (i.e., neutral). Likewise, the intensity of the emotional excitement perceived varied across the tasks and between the subjects (for details of the self-reports of each subject, please see Supplemental Material T1). Importantly, the intensity reported in self reports is not predictive of the emotional expressions shown. For example, even though fear is reported as the second emotion per high level of intensity, the number of the clips is relatively low compared to other emotions (e.g., happiness). Moreover, not all subjects display the entire range of emotions. While happiness and disgust were easy to induce (see Fig. 2), other emotions such as fear and anger were challenging to elicit (possible theoretical interpretations for these results are provided in the Section “Discussion”).

## Validation

### Participants

Being the number of stimuli very high (*N* = 1731), they were split into four independent blocks, each of them including approximately 400 stimuli. Each rater was randomly assigned to one block. A total of 122 participants were recruited for the validation study and were matched for age (mean = 25.3; SD = 2.47) and gender (male = 58; female = 64), with the participants included in the first part of this study (i.e., “actors”). The validation group included 58 males and 64 females. Each block was validated by 30 independent raters. No between-group differences in age or gender were present. A further 29 subjects did open the link to the rating task but never started it (i.e., 23.8% drop-out). Of all 122 participants, 98 (80.3%) completed the entire rating, while 24 raters (19.7 %) did not. Among these, 25% (six out of 24) completed more than 70% of the questionnaire. The rest of participants (18 out of 24) partially rated the validation (23.8% on average), and their data is included. Participants were all graduate students at the University of Padova (Italy). The majority of the participants were recruited through the institute’s participant pool. Others were recruited from online University discussion forums.

### Validation procedure

The validation procedure was sent online to participants’ email addresses using Qualtrics software (http://www.qualtrics.com). Participants were shown short clips displaying facial expressions of anger, disgust, fear, happiness, sadness, and surprise from the PEDFE. During the validation session, the original audio was removed from the video, in order to avoid the results on emotion recognition and genuineness to be inflated by the presence of the audio. The validation was conducted according to Dawel et al., (2017). After each of the emotional clips, participants were asked to categorize the emotion (they have to choose one within the six basic emotions, or neutral, or other, to give them the possibility to indicate an emotion not included within the six basic ones (Frank and Stennett, 2001), and the type of expression (i.e., genuine or fake, on a Likert scale ranging from -7 -not genuine at all- to 7 – totally genuine- ; Dawel et al., 2017) displayed. The neutral midpoint “0” corresponded to “I do not know”. This method allowed us to assess the ratings in absolute terms (i.e., genuine or fake). Furthermore, it provided information regarding the gradient of genuineness perceived by raters (e.g., + 7 indicates that the emotion was perceived as genuine without any doubt by the observer, a different gradient from a score of + 1, very close to “0”). Last, participants evaluated how intense the observed emotions looked to them on a Likert scale ranging from 0 (no intense at all) to 9 (extremely intense) (Dawel et al., 2017).

Regarding the emotion recognition, we calculated the “hit rates” by dividing the number of accurately recognized emotions by the total number of displays for that emotion. Regarding genuineness recognition, we calculated the “hit rate” of genuineness by dividing the number of accurately recognized emotions as genuine or posed by the total number of displays. Simultaneously, the Mean and the Standard Deviation (SD) of the gradient of genuineness were also calculated. Finally, the mean and SD of perceived intensity were calculated for each clip. The questionnaire took about 2 h and 30 min to be completed. However, participants were strongly suggested to divide the questionnaire into three days (i.e., 45 min of task per day).

### Validation results

**Fig. 5:**
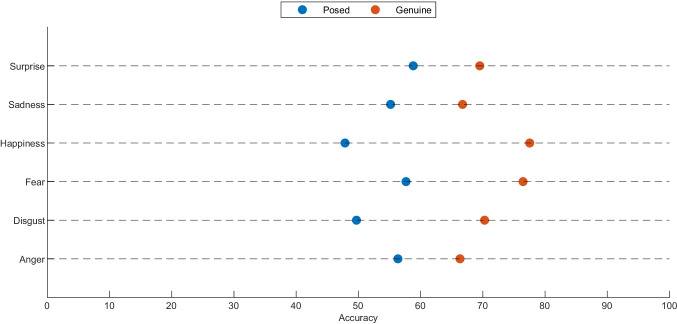
Genuineness hit rate for each emotion

The “hit rate for emotion” was adopted as the main exclusion criteria for the original 1731 clips. In fact, all the clips recognized with a “hit rate for emotion” less than 30% were removed from the entire dataset, obtaining 1458 emotional clips (i.e., 707 spontaneous and 751 posed) in total. The list of the final stimuli, including the hit rates for emotion and genuineness, intensity and genuineness rating, as well as the duration of each clip is provided in Supplemental Material T2 which is available at the OSF page provided within the “availability” section at the end of this manuscript. In Table 2, the total number of clips included in PEDFE, as well as the hit rates, divided for emotion (e.g., disgust) and genuineness (i.e., spontaneous and posed), are reported respectively. Furthermore, the same analysis was conducted more in detail for every single subject actor included in the PEDFE’s clips (please see Supplemental Material T7). Notably, on average, regardless of genuineness (i.e., spontaneous or posed), all the emotions were categorized with an accuracy of 78.6%, ranging from 58.01% (for fear) to 93.66% (for happiness). As expected, happiness is the best-labeled emotion (both for spontaneous and posed expressions). Conversely, fear is the worst in accordance with the literature that reveal lower recognition rates of fear than the other basic emotions (Roy-Charland et al., 2014). Further analyses were run in order to investigate if the cause of the low accuracy rating of fear was due to the misclassification with the surprise. To do this, we calculate the number of times the emotion was categorized as a surprise for each clip. Results confirmed that, on average, fear is labeled as a surprise 29.76% of the time (SD 19.71%). Additionally, to evaluate if the intensity perception of the emotional expressions affects the emotion’s discrimination, we conducted the Pearson correlation test. Importantly, the hit rate seems to be moderately affected by the intensity of the emotions expressed (*r* = 0.44, for 1458 items), in particular for anger expressions (*r* = 0.67 for 166 items). The correlations between hit rate per emotion and intensity are reported in Supplemental Material T4 for each emotion. For what concerns the hit rate for the genuineness categorization, the global accuracy is stable across all the emotions (i.e., 62.51%), ranging from 60.22% (for disgust) to 65.25% (for fear). More precisely, genuine emotions were categorized better (i.e., 71.92% on average) than the posed ones (i.e., 53.65% on average), regardless of the emotion displayed (please see Fig. 5). Chi-squared test among all the binary responses extract by raters for each emotional stimulus confirmed the significant effect of the type of the stimuli (i.e., spontaneous or posed) on the hit rate of genuineness for each emotion with a *p* < 0.00001. In particular, anger *χ*^2^(1,*N* = 4662) = 100.65, *p* < 0.00001, disgust *χ*^2^(1,*N* = 7719) = 221.97, *p* < 0.00001, fear *χ*^2^(1,*N* = 4049) = 164.53, *p* < 0.00001, happiness *χ*^2^(1,*N* = 10876) = 376.52, *p* < 0.00001, sadness *χ*^2^(1,*N* = 6619) = 172.65, *p* < 0.00001, and surprise *χ*^2^(1,*N* = 5823) = 100.94, *p* < 0.00001. In other words, people tended to classify posed emotions as genuine more often than they classify genuine as posed. Differently from the hit rate for emotion, these results are completely unrelated to the intensity (*r* = 0.11, for 1458 item) or the emotion (*r* = 0.06, for 1458 item) expressed. A theoretical explanation of these results is provided in Section “Discussion”.

To compare the intensity rates between genuine and not genuine emotions for each emotion expressions, a factorial ANOVA was run using genuineness (two levels: genuine, posed) and emotions (six levels: the six emotions) as independent variable and intensity rates as dependent variables. The analyses revealed a trend toward significance for the main effect of Genuineness (*F*[1,1446] = 3.62,*p* = 0.057) as posed emotions are generally rated as more intense than genuine ones (3.95 vs. 3.75). A significant main effect of emotions was also observed (*F*[5,1446] = 30.62,*p* < 0.001), where fear was the emotions rated generally as more intense (4.31), followed by disgust (4.15, *p* = 0.11 when compared with fear), and happiness (4.04, *p* = 0.02 when compared with fear and *p* = 0.27 when compared with disgust). Then, the emotion of surprise (3.73, *p* < 0.001 compared with all the other emotions), anger (3.42, *p* < 0.001 compared with all the other emotions except sadness, where *p* = 0.24) and sadness (3.30, *p* < 0.001 compared with all the other emotions except anger). Critically, the interaction genuineness x emotion is significant as well (*F*[5,1446] = 9.50, *p* < 0.001). Newman–Keuls post hoc test, revealed that intensity did not differ between genuine and posed disgust (4.10 vs. 4.20, *p* = 0.53), between genuine and posed fear (4.48 vs. 4.20, *p* = 0.12) and between genuine and posed anger (3.37 vs. 3.46, *p* = 0.83). Contrarily, genuine happiness and sadness are perceived as more intense than their posed counterpart (4.31 vs. 3.66, *p* < 0.001 for happiness and 3.52 vs. 3.09, *p* = 0.022 for sadness), while genuine surprise is perceived as less intense than posed one (3.41 vs. 3.92, *p* = 0.005).

**Table 2:** Total number of clips included in PEDFE, followed by their respective hit rates

	TOT	POS	GEN	HR Emo TOT (%)	HR Emo POS (%)	HR Emo GEN (%)	HR Type TOT (%)	HR Type POS(%)	HR Type GEN (%)
Anger	166	90	76	64.88	69.30	59.64	60.92	56.36	66.33
Disgust	305	149	156	84.48	87.10	81.98	60.22	49.69	70.28
Fear	156	93	63	58.01	53.95	64.01	65.25	57.66	76.47
Happiness	370	156	214	93.66	93.42	93.84	65.02	47.85	77.53
Sadness	251	132	119	71.09	73.57	68.35	60.66	55.18	66.74
Surprise	210	131	79	78.70	85.44	67.52	62.84	58.81	69.51
ALL	1458	751	707	78.61	79.51	77.66	62.51	53.65	71.92

TOT: Total number of clips; GEN: Number of Genuine clips; POS: Number of Posed clips; HR Emo TOT: Emotion hit rate for the total number of clips; HR Emo POS: Emotion hit rate for Posed clips; HR Emo GEN: Emotion hit rate for Genuine clips; HR Type TOT: Genuineness hit rate for the total number of clips; HR Type POS: Genuineness hit rate for Posed clips; HR Type GEN: Genuineness hit rate for Genuine clips

## Creation and validation of the modified version of the dataset

### Video enhancement

**Fig. 6:**
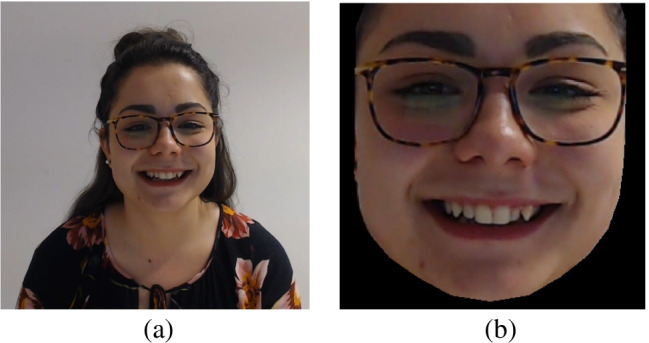
Clip pre- and post-production

After all the emotional facial expressions were rated from the entire validation, the clips surviving the validation (*N* = 1458) were submitted through different video processing steps. These phases aim to obtain clips containing only the face of the participant, removing everything that did not strictly concern facial expression. First, the clips were processed using OpenFace (Baltrušaitis et al., 2016). OpenFace is a face detection software based on deep neural networks that we used to extract for each clip frame containing only the face of the subject (i.e., the background was removed, see Fig. 6).

**Fig. 7:**
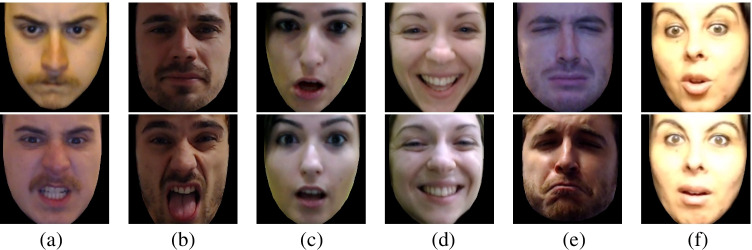
Peak intensity images of genuine (*first row*) and posed expressions (*second row*) of the six emotions included in PEDFE

The size of each frame is fixed and was manually set to 300 x 300 pixels, meaning that all the extracted faces were resized to fit these constraints. In addition, OpenFace provides bidimensional coordinates of 68 facial landmarks for each frame. To maintain the native dimension of the faces, in order to avoid stretched images, we leveraged the coordinates of the landmarks to resize the frames of each clip. In particular, the maximum difference among *x*-coordinates and *y*-coordinates per frame was extracted. We then calculated the median value among all the frames of a clip, obtaining the native size of each face. Finally, we resized each frame of a clip to the corresponding native size, and we padded the frame with black pixels, obtaining new clips of 854 x 480 pixels (see Fig. 7). Moreover, for each clip, the pictures captured frame by frame displaying the emotions’ temporal dynamics are also provided, except for the clips “5_dg_1” and “30_dg_1” that were successively removed due to the low quality of the recordings. The pictures were included in the dataset available to the scientific community as they can be beneficial to researchers to investigate the course of the emotional expression as well as the various degrees of intensity of the emotions (e.g., from neutral to mid to high intensity) with static pictures.

### Enhanced videos validation procedure

The validation procedure was the same already described for the original clips, with the exception that it was run on 1458 clips divided in four blocks (two blocks including 364 stimuli and two blocks including 365 stimuli). Each block was validated by 70 independent raters for a total of 280 raters (mean age 24.87, SD 2.56 – 132 M and 148 F). All the raters fully completed the assigned block.

### Enhanced videos validation results

**Fig. 8:**
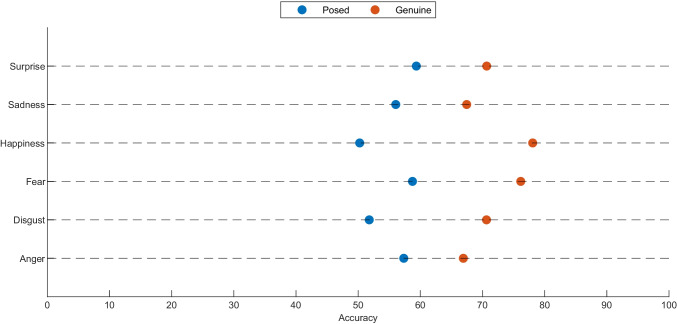
Genuineness hit rate for each emotion for the enhanced dataset

For the enhanced dataset, Supplemental Material T5 contains the list of stimuli, including their hit rates for emotion, intensity, and genuineness. The number of clips included in the enhanced dataset is reported in Table 3, as well as the hit rates for anger and sincerity. Furthermore, the same analysis was conducted more in detail for every single subject actor included in the enhanced dataset’s clips (please see Supplemental Material T7). Notably, on average, regardless of genuineness (i.e., spontaneous or posed), all the emotions were categorized with an accuracy of 79.07%, ranging from 58.95% (for fear) to 93.82% (for happiness). In addition, to determine whether perception of the intensity of emotional expression influences its discrimination, we conducted a Pearson correlation test. Also for the enhanced dataset, the hit rate seems to be moderately affected by the intensity of the emotions expressed , in particular for anger expressions (r = 0.62 for 166 items). The correlations between hit rate per emotion and intensity are reported in Supplemental Material T6 for each emotion. According to the global accuracy measure for genuineness categorization, the hit rate is stable across all the emotions (i.e., 63.50%), ranging from 61.43% (for disgust) to 66.34% (for happiness). In particular, genuine emotions were categorized better (i.e., 72.45% on average) than the posed ones (i.e., 55.05% on average), regardless of the emotion displayed (please see Fig. 8). Chi-squared test among all the binary responses extract by raters for each emotional stimulus confirmed the significant effect of the type of the stimuli (i.e., spontaneous or posed) on the hit rate of genuineness for each emotion with a *p* < 0.00001. In particular, anger *χ*^2^(1,*N* = 11620) = 104.18, *p* < 0.00001, disgust *χ*^2^(1,*N* = 21350) = 798.23, *p* < 0.00001, fear *χ*^2^(1,*N* = 10920) = 361.14, *p* < 0.00001, happiness *χ*^2^(1,*N* = 25900) = 2203.88, *p* < 0.00001, sadness *χ*^2^(1,*N* = 17570) = 228.14, *p* < 0.00001, and surprise *χ*^2^(1,*N* = 14700) = 186.16, *p* < 0.00001.

To compare the intensity rates between genuine and not genuine emotions for each emotion expressions, a factorial ANOVA was run using genuineness (two levels: genuine, posed) and emotions (six levels: the six emotions) as independent variable and intensity rates as dependent variables. The analyses revealed a significant main effect of Genuineness (*F*[1,1446] = 3.96, *p* = 0.046) as posed emotions are generally rated as more intense than genuine ones (4.06 vs. 3.86). A significant main effect of emotions was also observed (*F*[5,1446] = 27.75, *p* < 0.001), where fear was the emotions rated generally as more intense (4.38), followed by disgust (4.23, *p* = 0.16 when compared with fear), and happiness (4.16, *p* = 0.10 when compared with fear and *p* = 0.52 when compared with disgust). Then, the emotion of surprise (3.88, *p* < 0.001 compared with all the other emotions), anger (3.50, *p* < 0.001 compared with all the other emotions except sadness, where *p* = 0.48) and sadness (3.43, *p* < 0.001 compared with all the other emotions except anger). Critically, the interaction genuineness x emotion is significant as well (*F*[5,1446] = 10.18, *p* < 0.001). Newman–Keuls post hoc test, revealed that intensity did not differ between genuine and posed disgust (4.19 vs. 4.28, *p* = 0.83), between genuine and posed fear (4.58 vs. 4.24, *p* = 0.10) and between genuine and posed anger (3.46 vs. 3.53, *p* = 0.60). Contrarily, genuine happiness is perceived as more intense than its posed counterpart (4.46 vs. 3.76, *p* < 0.001), while genuine surprise is perceived as less intense than posed one (3.55 vs. 4.09, *p* = 0.002). Finally, a trend toward significance emerges toward a more intense genuine sadness (3.63) than the posed one (3.24, *p* = 0.06). In conclusion, the results of the validation on the enhanced dataset are perfectly in line with the results of the original dataset.

**Table 3:** Total number of clips included in the enhanced dataset, followed by their respective hit rates

	TOT	POS	GEN	HR Emo TOT (%)	HR Emo POS (%)	HR Emo GEN (%)	HR Type TOT (%)	HR Type POS(%)	HR Type GEN (%)
Anger	166	90	76	65.41	69.60	60.44	61.72	57.34	66.92
Disgust	305	149	156	84.94	87.49	82.51	61.43	51.78	70.65
Fear	156	93	63	58.95	54.94	64.87	65.70	58.74	76.15
Happiness	370	156	214	93.82	93.60	93.98	66.34	50.25	78.08
Sadness	251	132	119	71.67	74.05	69.03	61.48	56.05	67.46
Surprise	210	131	79	79.12	85.82	68.00	63.64	59.36	70.68
ALL	1458	751	707	79.07	79.93	78.15	63.50	55.05	72.45

TOT: Total number of clips; GEN: Number of Genuine clips; POS: Number of Posed clips; HR Emo TOT: Emotion hit rate for the total number of clips; HR Emo POS: Emotion hit rate for Posed clips; HR Emo GEN: Emotion hit rate for Genuine clips; HR Type TOT: Genuineness hit rate for the total number of clips; HR Type POS: Genuineness hit rate for Posed clips; HR Type GEN: Genuineness hit rate for Genuine clips

## Discussion

So far, the emotions conveyed by faces classically used as emotional stimuli in the research on emotions are not genuine. Thus, to date it is still unknown whether our actual knowledge on perception of emotions conveyed by faces is biased by the unconscious perception of the non-authenticity of the emotion expressed and thus, if results achieved so far could be generalized to the perception of authentic, more ecological, expressions. The current work aims to provide the scientific community with a new dataset of emotional facial expressions including both spontaneous (i.e., genuine) and posed emotions from the same actor and validated by independent raters. The dataset is available in two versions: original and modified, where the modified dataset includes only faces without the background. Genuine emotions were elicited using an innovative multi-modal elicitation strategy, that allowed us to select the most effective strategy for each emotion’s peculiarity. In the final dataset, which includes 707 spontaneous and 751 posed emotions, facial expressions of the six basic emotions are displayed both in dynamic clips and static pictures. As expected, some emotions such as fear and anger were more challenging to elicit than others (e.g., happiness or disgust) and, as a consequence, the number of stimuli included in the dataset varies according to the emotion expressed. For example, PEDFE contains 370 clips of happiness expressions and “only” 156 of fear and 166 of anger. This finding is perhaps not surprising, considering that fear and anger are known as the most difficult emotions to elicit (Rottenberg et al., 2007). The reason why anger is difficult to elicit might be because anger requires a high level of personal engagement to be experienced (Zupan & Babbage, 2017). The vision of clips and the rage game used in the elicitation protocol might have not triggered high levels of anger in all participants. As with regard of fear, this emotion was in some participants expressed through a passive freezing reaction (Lojowska et al., 2018; Roelofs, 2017), which was translated in a subjective experience of fear in the absence of facial movements. This made the detection and recognition of fear by means of facial clues harder. In addition, stimuli aiming to elicit both anger and fear often cause a blend of negative emotions, such as disgust and sadness in the case of anger, or tension and anxiety in the case of fear (Rottenberg et al., 2007).This likely contribute to the expression of mixed emotions, not surviving to the stringent selection strategy we adopted, consisting in matching the emotion subjectively felt by the participants (rating), with the emotions expressed and codified by a certified FACS expert. This of course contributed to the relatively low number of clips. In general, regardless of the emotion considered, the collection of spontaneous expressions in an experimental setting is not easy because of a trade-off between ecological reactions and methodological restrictions (Sneddon et al., 2011; Tcherkassof et al., 2013). To make sure that participants’ emotional facial expressions were natural and spontaneous, no restrictions (e.g., movements, eye gaze, the intensity of the expressions) were given to participants. This choice inevitably made it impossible to match the number of genuine and posed emotions perfectly. Furthermore, the great variability among the participants’ sensitivity affected the expressions of emotions both between subjects and within the same subject (i.e., in expressing spontaneous and posed emotions). However, this limitation offers, at the same time, an ecological set of spontaneous facial expressions, providing emotions that differ under different features, such as the intensity of the expression, eye gaze, head movements.

Another contribution comes from the elimination of the background. Indeed, all the incidental features such as hair, clothes, the color of the setting room that may influence emotional expression perception were removed from the background of the stimuli. In other words, only the face on a black screen was portrayed in the clips. As emerged from the validation of the enhanced dataset, recognition of emotion and genuineness, as well as intensity rating by perceivers, are not significantly affected by the modification applied to the original clips A further significant benefit of the isolation of the background concerns the automatic detection of the emotional facial expressions. Indeed, many face recognition algorithms require prior segmentation and alignment or faces, failing with non-uniform background. Isolating the face from the background can help the algorithms align the face to a standard template and improve facial expressions’ accurate detection (Tsao & Livingstone, 2008). Notably, all stimuli were validated by human observers. The normative data obtained are in line with the typical finding in expressions databases (Langner et al., 2010; Palermo & Coltheart, 2004). More precisely, the hit rate for emotion is, on average, more than 93% for happiness and ranging from 64.88 to 84.48% for the other emotions. The only exception is fear, where the hit rate for emotion is 58.01%. However, it is widely known that fear is easily mistaken for a surprise (Ekman, 1976; Rapcsak et al., 2000; Ekman & Friesen, 1971; Wang & Markham, 1999). The low level of accuracy in fear was indeed due to this typical tendency. In general, the emotion accuracies are moderately correlated with the intensity of the emotion perceived as reported in section Supplemental Material T4 (for the original dataset) and T6 (for the enhanced dataset). In other words, the more intense the emotion is expressed, the higher is the accuracy rate for the emotion, in accordance with the literature of emotions. It is known how low intensity reduces labeling accuracy, affecting the observers’ ability to detect whether or not an expression is shown because of insufficient physical information in the face (O’Reilly et al., 2016; Barrett et al., 2019; Dawel et al., 2017). Different from the hit rate for emotion, the accuracy of the hit rate of genuineness is on average 62.51%, highlighting the difficulties manifested by humans in genuineness recognition . In fact, it is known that people (both untrained observers and professional experts like psychologists) are unable to discriminate genuine from not genuine emotional displays, in particular, if they have to rely on visual cues only (Bartlett et al., 2006). Several studies demonstrated how people tend to perform not far from the chance level when asked to detect such behaviors (Porter & Ten Brinke, 2008; Porter et al., 2012; Vrij, 2008; Levine et al., 1999; Porter & ten Brinke, 2010). Furthermore, this problem is amplified by people’s tendency to believe that the person with whom they are speaking is honest, regardless of whether or not that person is lying or being untruthful (Levine, 2014; McCornack & Parks, 1986). This mechanism called truth-bias belongs to human nature to believe and weakens its ability to detect deception. This was also confirmed in the validation of PEDFE, where the hit rate for the genuineness of posed emotion (i.e., when participants should have classified emotions as posed to respond correctly) is on average 53.65%. Conversely, the hit rate for the genuineness of genuine emotion (i.e., when participants should have classified emotions as genuine to respond correctly) is 71.92%. Also note, these results do not change according to the intensity of the emotion expressed. In other words, the intensity of the expression does not improve the accurate detection of spontaneous and posed emotional facial expressions differently for the hit rate for emotion.

## Conclusions

This paper presents a new validated dataset of facial expressions displaying spontaneous and posed emotions. PEDFE contributes a unique source of ecological stimuli, providing 1458 dynamic clips and the pictures frame by frame of each stimulus. The significant amount of emotions included in PEDFE, offers an excellent choice and a vivid picture of the variability in emotional expressions permeating real-life situations. Furthermore, the normative data give insight into the perception of emotional facial expressions by human observers. PEDFE may be an invaluable resource in different fields of study, such as psychology and analysis of non-verbal behavior, affective computing, and emotional lie detection. Future works will aim to enrich the dataset with new participants and more complex emotions.
